# Construction and validation of an infant chest phantom for paediatric computed tomography

**DOI:** 10.1007/s13246-023-01379-5

**Published:** 2024-02-05

**Authors:** Seonaid Rodgers, Janette Atkinson, David Cryer, Cameron Storm, Rikki Nezich, Martin A. Ebert, Pejman Rowshanfarzad

**Affiliations:** 1 Department of Medical Technology and Physics, Sir Charles Gairdner Hospital, Hospital Avenue, Nedlands, WA 6009 Australia; 2 School of Physics, Mathematics and Computing, University of Western Australia, Crawley, WA Australia; 3 Department of Radiation Oncology, Sir Charles Gairdner Hospital, Nedlands, WA Australia; 4 Centre for Advanced Technologies in Cancer Research (CATCR), Perth, WA Australia

**Keywords:** Paediatric, Chest, Phantom, Diagnostic, Radiology, Computed tomography

## Abstract

Paediatric imaging protocols should be carefully optimised to maintain the desired image quality while minimising the delivered patient dose. A paediatric chest phantom was designed, constructed and evaluated to optimise chest CT examinations for infants. The phantom was designed to enable dosimetry and image quality measurements within the anthropomorphic structure. It was constructed using tissue equivalent materials to mimic thoracic structures of infants, aged 0–6 months. The phantom materials were validated across a range of diagnostic tube voltages with resulting CT numbers found equivalent to paediatric tissues observed via a survey of clinical paediatric chest studies. The phantom has been successfully used to measure radiation dose and evaluate various image quality parameters for paediatric specific protocols.

## Introduction

Children are known to be more susceptible than adults to the harmful effects of ionising radiation due to their highly proliferating cells and longer life expectancy [1, 2]. Consequently, paediatric Computed Tomography (CT) referrals need to be thoroughly justified [3] and associated CT dose should be minimised whilst still providing image quality sufficient for purpose. Several dose reduction techniques are available on modern CT scanners such as the automatic tube current modulation [4] and more recently, added tin filtration available on the Somatom Force clinical CT scanner (Siemens Healthcare, Forchheim, Germany) [5].

Phantoms play an essential role in quality assurance of diagnostic imaging systems. Typically, phantoms used in radiology physics are purpose built for either dosimetry or image quality tests [6]. The standard computed tomography dose index (CTDI) phantoms are used to estimate paediatric dose; however, they contain no image quality tools. Standard, adult anthropomorphic phantoms [7, 8] are not appropriate for paediatric dose optimisation due to significant differences in anatomical shape, size and tissue composition and resulting CT numbers of anatomical structures, particularly bony structures and lung tissue. Studies [9, 10] have developed paediatric chest phantoms suitable for general radiography. Personalised anthropomorphic, phantoms have been constructed based on newborn CT scans using both advanced moulding techniques [6, 11] and 3D printing technology [12]. Various commercial paediatric phantoms are available [13–15] but costly.

In this study, a paediatric chest phantom was designed and constructed using tissue-equivalent materials for the purpose of simulating the radiological properties of an infant during a chest CT examination. This low-cost design enables simultaneous measurements of dose and image quality. The present work outlines the methods undertaken to construct and validate this phantom.

## Methods

### Tissue substitute materials

Three foundational tissues were required: soft tissue, lung tissue and a bony substitute for ribs and spine. The selection of tissue substitute materials was based on their availability, cost, physical properties and radiological properties. Radiological properties were evaluated through pixel value analysis of reconstructed CT images of samples scanned at various tube voltage and filtration combinations. The CT number of candidate materials were compared to the measured CT numbers from tissues within a clinical survey of paediatric tissues (CSPT) to validate tissue-equivalent materials.

### Phantom design and construction

**Fig. 1:**
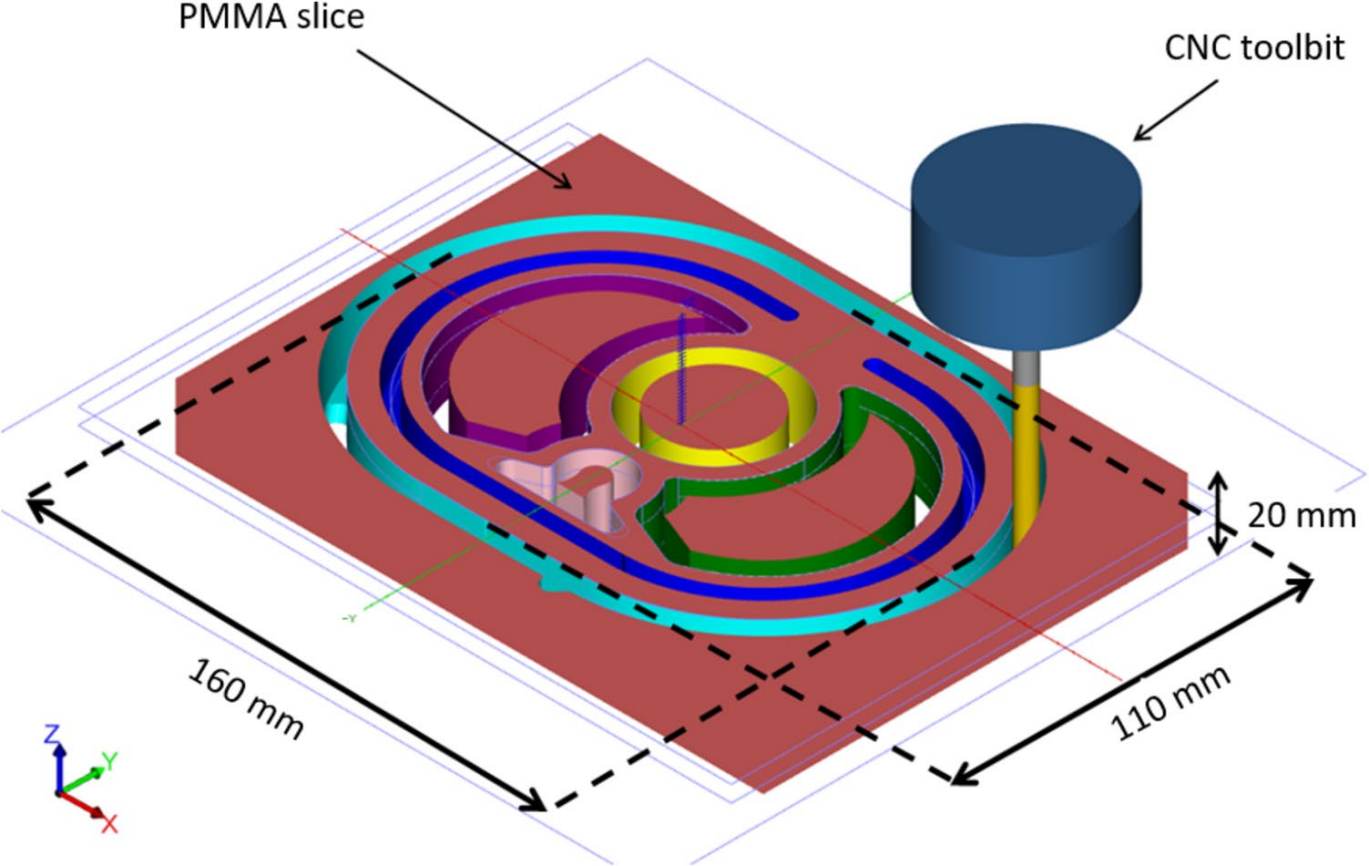
3D illustration of the phantom slice design in MasterCam to be machined via CNC. Cavities for organs are illustrated by coloured tool pathways: central rod cavity (yellow), lung cavities (purple and green), spine (light pink), ribs (dark blue) the skin and edge of the phantom slice (aqua). Note, the rib cavities were cut only halfway through each slice to create soft tissue separation between ribs

The phantom was designed in 20 mm thick, 160 mm $\times$ 110 mm axial slices held together by a central PMMA rod. Phantom slices and cavities for organs were designed on Mastercam and machined using a computer numerical controlled (CNC) milling machine. The basic anatomical design of the phantom slices is illustrated in Fig. 1. Cavities were cut 10 mm deep into the axial slice thicknesses to produce ribs.

The bone substitute was hand-mixed and poured into the spine and rib cavities (light pink and dark blue in Fig. 1). The lung substitute was cut to size using a bandsaw and inserted into the lung cavities. Three central rods, designed to impale any combination of eight slices, simulate soft tissue and model heart muscle. The original central rod was modified twice to allow for dosimetry capabilities.

### Dosimetry and image quality slices

**Fig. 2:**
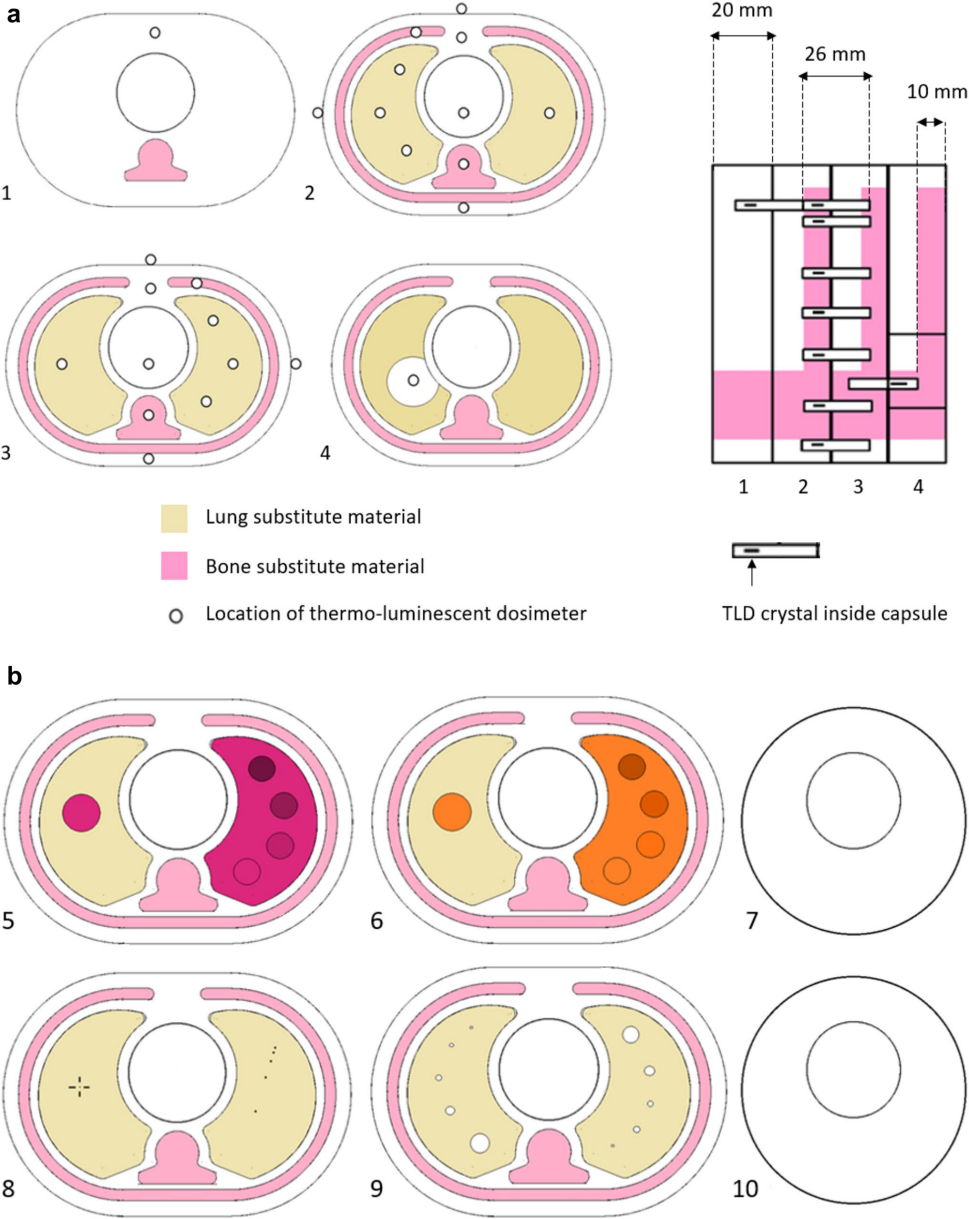
**a** (Left) Axial view of the dosimetry slices utilising holes for TLDs. The superior slice (1), contains a cavity for a thyroid TLD. The two central anatomical slices (2 and 3) encompass an array of TLD positions to estimate dose delivered to skin, breast, ribs, spine and heart. The inferior slice contains an additional PMMA insert to estimate dose to the liver (4). Right) Lateral view of the dosimetry slices, pink indicating the bone substitute material running through the posterior of all slices (spine) and the repetitive pattern of 10 mm thick ribs within slices 2, 3 and 4. TLD capsules were positioned between adjacent phantom slices as the length of the capsules (26 mm) exceeds the phantom slice thickness (20 mm). **b** Six custom made image quality slices. Four anthropomorphic slices with image quality inserts for measuring contrast and resolution in soft tissue substitute (5) and lung substitutes (6), spatial resolution (8) contrast detail (9) and two slices for uniformity test (7 and 10)

The slices 1–4 were custom made for paediatric specific dosimetry using thermo-luminescent dosimeters (TLDs). Slices 5–10 were designed to simulate the radiological properties of an infant while encompassing image quality tools. The design for dosimetry slices are illustrated in Fig. 2a and the design for image quality slices are illustrated in Fig. 2b. A more detailed description of each phantom slice is given in Table 1.

**Table 1:** Description of each phantom slice

#	Slice	Purpose	Details of slice construction/modification
1	Superior dosimetry slice	Designed to model the apex of the infants’ lungs, shoulders and thyroid	Ribs and lungs were removed from anatomical slice. With the addition of a hole to accommodate TLDs for thyroid dosimetry
2 and 3	Central dosimetry slices	Designed to estimate organ dosimetry in the breast, ribs, spine and heart	Minimal modification from original anatomical slice design. Nine internal holes drilled for TLDs
4	Inferior dosimetry slice	Designed to model the base of the lungs including the liver	Cork was partially removed and replaced with a soft tissue PMMA insert including a TLD hole for liver dosimetry
5	Contrast resolution soft tissue slice	Designed to measure low contrast resolution in a soft tissue substitute material	A simple 20 mm diameter, soft tissue ROI was filled with soft tissue substitute. The substitute material was a 3-part silicone-based solution (Eurosil-4 Pink, SynTech). The other lung was completely replaced by contrast levels. Each contrast insert was Eurosil-4 mixed with increased amounts of a synthetic softener which decreased the HU of the inserts incrementally
6	Contrast resolution lung tissue slice	Designed to measure low contrast resolution in the lung tissue substitute material	The substitute material was low density polyurethane foam. A simple 20 mm diameter ROI was made and the opposite lung was completely replaced by four contrast levels. The contrast inserts were made by soaking the polyurethane foam in various concentrations of iodinated contrast solution which increased the HU incrementally
7 and 10	Two circular uniformity slices	Designed to measure HU and noise uniformity	Custom slices, machine cut from 20 mm thick PMMA sheets and polished to fit. Instead of the oblique shape of the anatomical slices, the uniformity slices are circular with 110 mm diameter
8	Spatial resolution slice	Designed to assess both spatial resolution and distance accuracy	Cork was replaced with EVA foam to reduce the inherent structural noise. A point source wire (0.23 mm diameter, Nichrome) used for measuring axial spatial resolution was inserted in the z direction of the lung. The same wire was used in the opposite lung to make a calliper tool. Five wires were used for the calliper tool with incremental (20 mm, 10 mm, 5 mm and 2.5 mm) spacing between the wires
9	Contrast detail slice	Designed for the subjective assessment of contrast detail	Both cork sections were modified with air gaps drilled with decreasing diameters 5 mm, 4 mm, 3 mm, 2 mm, and 1 mm. The pattern was reversed in each lung, to avoid any asymmetry effects of anatomical structures

### Phantom validation

Validation consisted of a comparison of CT numbers measured from the constructed phantom materials and the clinically observed CT numbers from the CSPT. A successful validation required that the measured CT number fall within the clinically observed range. It is important to note that fatty tissue was analysed during the clinical survey but was not modelled in the paediatric phantom; and therefore, not included in the validation assessment.

### Clinical survey of paediatric tissues (CSPT)

To establish an acceptable range of CT numbers for paediatric tissues a survey of paediatric chest studies was conducted. This survey was performed retrospectively on non-contrast, helical chest studies acquired on a Somatom Force clinical CT scanner (Siemens Healthcare, Forchheim, Germany), located at Perth Children’s Hospital, Western Australia, between January 2019 and April 2020. Patients, all aged between 0–2 years were grouped into four groups according to the scanning X-ray tube voltage and filtration combinations; 70 kVp, 80 kVp, 100 kVp, and 100 kVp with added tin filtration (Sn 100 kVp).

CT number analysis was performed on the Agfa Impax 6.7.0.6011 DICOM viewing system (Agfa Healthcare, Mortsel Belgium). Thoracic structures, ribs, spine, soft tissue, fat and lung were sampled using ROI of approximately 20mm^2^ in size, where possible. HU values were sampled across multiple axial slices within the thorax. The maximum and minimum CT numbers were recorded to communicate the entire clinical range observed in the paediatric structures rather than an average CT number.

## Results

### Tissue substitute materials

Polymethyl-methacrylate (PMMA) was selected as the soft tissue substitute due to its water equivalent properties at diagnostic energies [17] it’s cost-effectiveness, accessibility and durability.

Cork was selected for the lung tissue substitute due to its low density, affordability, accessibility and the resulting CT number falling within the clinical range of paediatric lung tissue found in the CSPT.

**Fig. 3:**
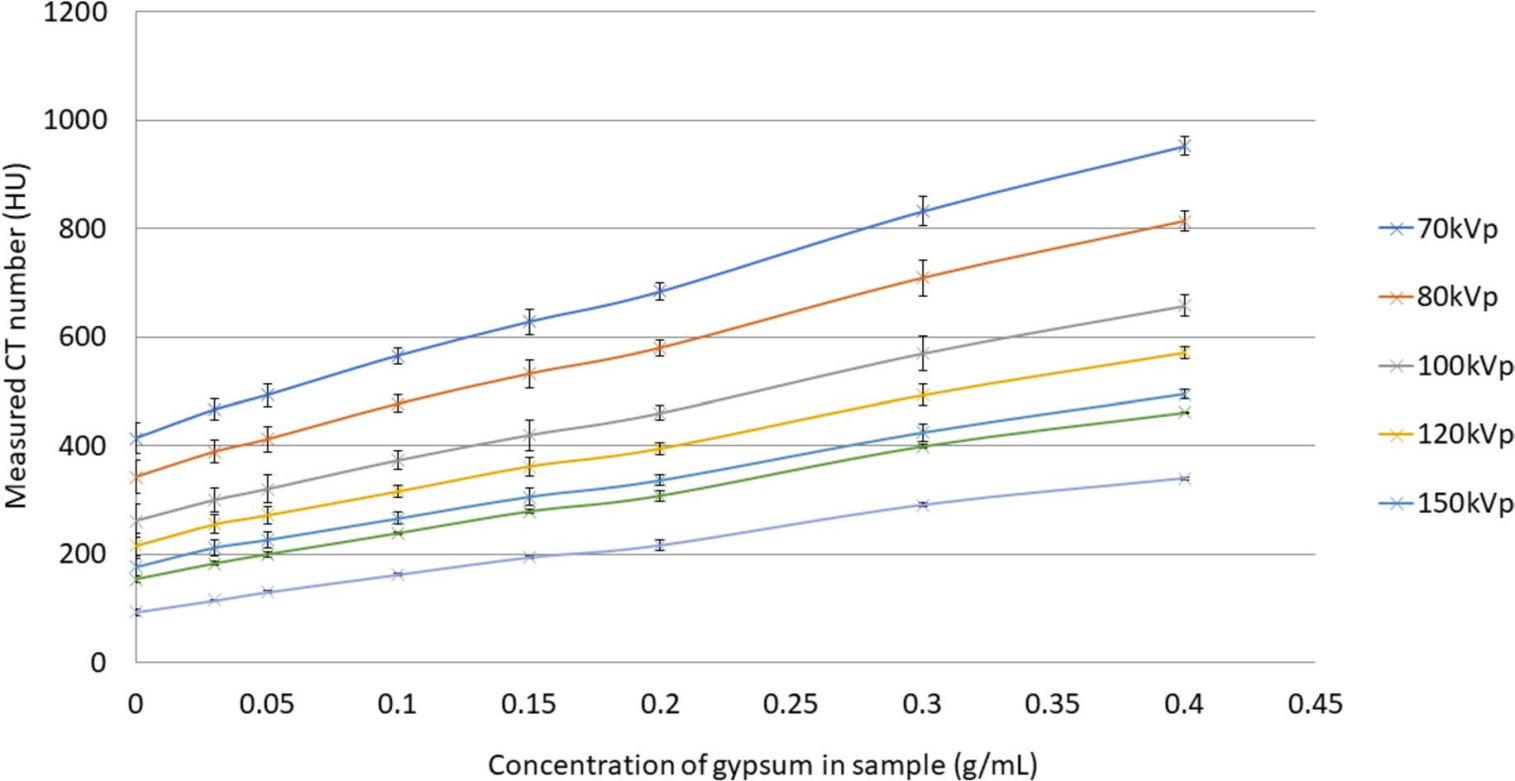
Results from bone substitute investigations. Resulting CT Number measured in Eurosil 10 solution and associated concentration of gypsum, measured at various tube voltage and filtration settings. Error bars indicate the range of CT numbers measured within each test sample

The bone substitute material chosen was Eurosil-10 Orange (SynTech, Nedtherlands), a silicone-based rubber solution mixed with added calcium sulphate dihydrate (gypsum) to produce the desired density and CT number. Figure 3 demonstrates the resulting CT numbers due to increasing concentrations of gypsum, evaluated across a range of diagnostic beam qualities in CT. This data was used together with the results from the CSPT to determine the desired formulation for paediatric spine and ribs. The concentrations of gypsum in Eurosil-10 solution selected to model the ribs and spine were 0.4 g/ml and 0.3 g/ml, respectively.

### Phantom design and construction

**Fig. 4:**
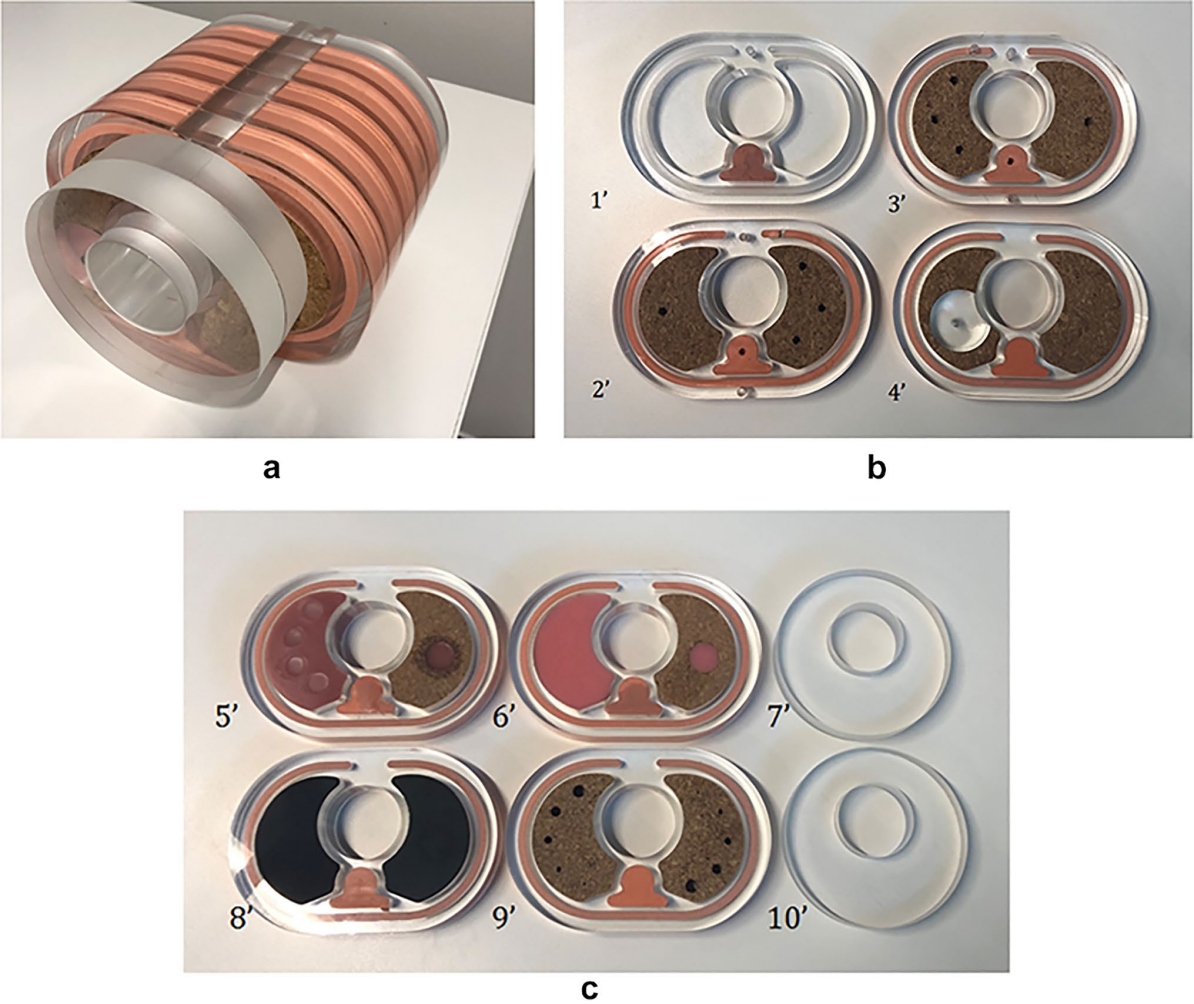
**a** Fully assembled phantom; 2 circular uniformity slices followed by 6 anatomically shaped slices for both dosimetry and image quality measurements. **b** Constructed dosimetry slices: Four anthropomorphic phantom slices with holes drilled for TLDs, corresponding to the designed dosimetry phantom slices in Fig. 2a. **c** Constructed image quality slices: Four anthropomorphic slices and two uniformity slices, corresponding to the designed image quality phantom slices in Fig. 2b

A total of 12 slices were fabricated, comprising of four dosimetry slices, six image quality slices and two spare anatomical slices. Three central rods: a standard 16 cm solid PMMA rod, a small volume dosimetry rod and a three-piece TL dosimetry rod. Any of these central rods can be used to assemble the phantom, accommodating a maximum of any 8 slices. Figure 4a shows a fully assembled phantom for reference.

### Dosimetry and image quality slices

The four constructed dosimetry slices containing TLDs are shown in Fig. 4b. For TL dosimetry, these slices are paired with the three-piece rod which provides access to a central TLD while also holding the slices together. The six image quality slices are shown in Fig. 4c, these slices can be arranged in any desired order on any central rod.

**Fig. 5:**
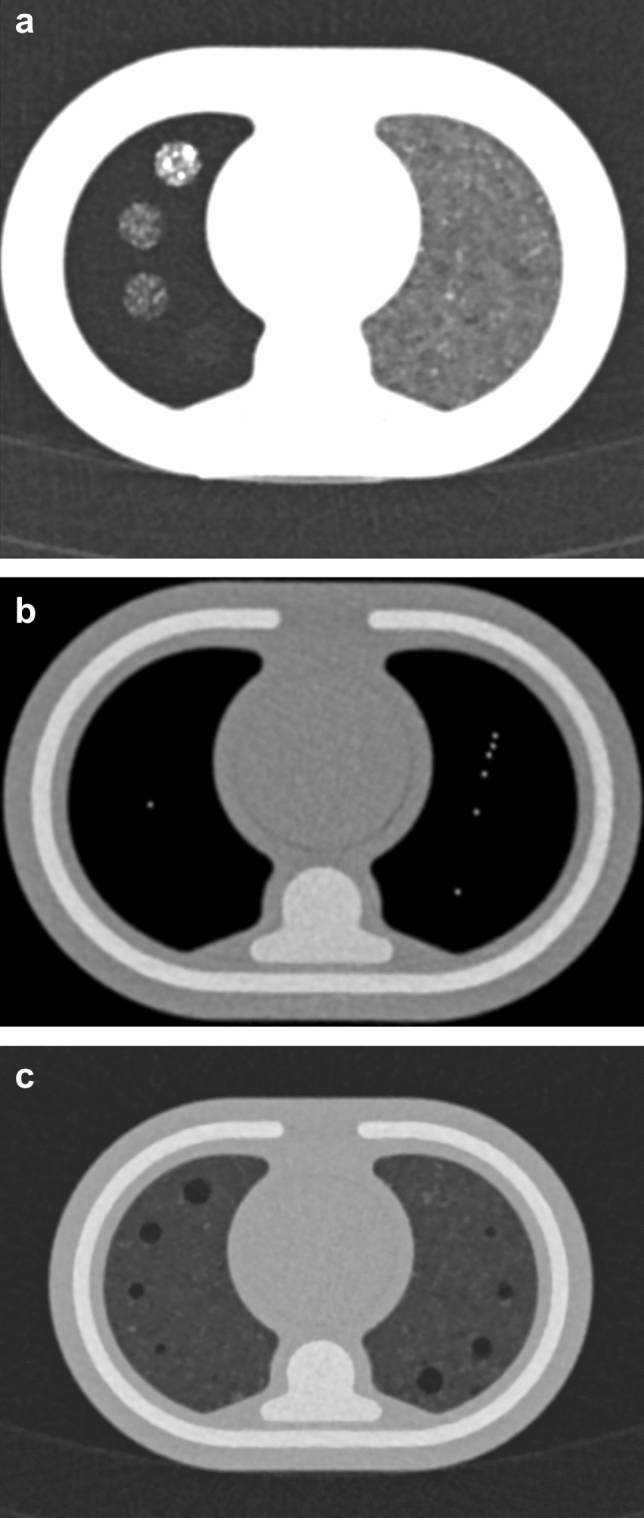
Reconstructed axial CT slices of image quality slices 6, 8 and 9: **a** Slice 6, the contrast resolution tool showing iodinated contrast levels inside the lung substitute. **b** Slice 8, the spatial resolution slice, showing thin Nichrome wire point source used to produce the MTF (left), and a 6-point calliper tool (right). **c** Slice 9, the contrast detail test tool with gradually smaller air gaps

**Fig. 6:**
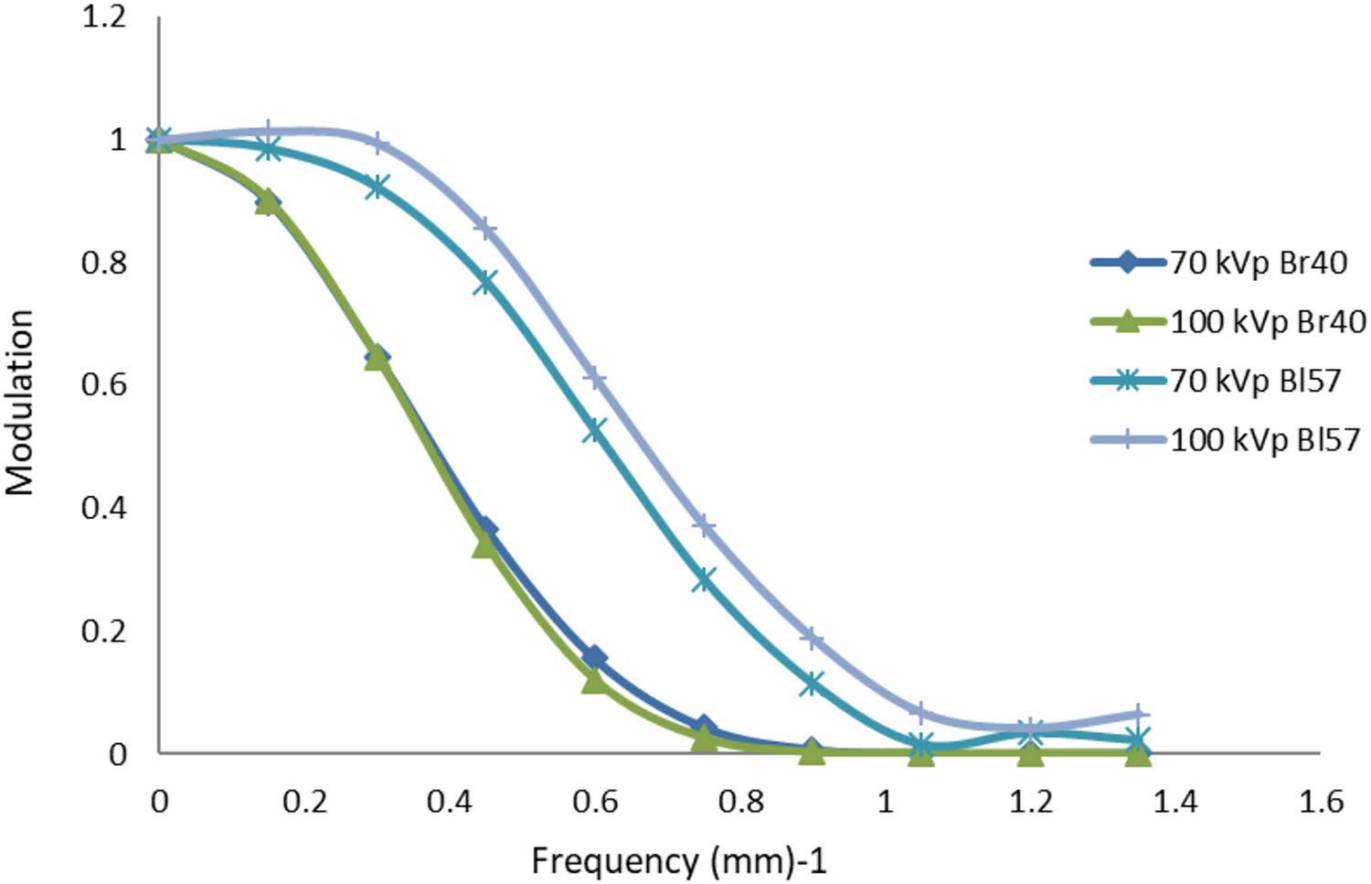
MTF’s created by the Nichrome wire point source signal in slice 8. The MTF comparisons at tube voltages 70 kVp and 100 kVp on both smooth and sharp reconstructed images

Figure 5 shows the reconstructed axial images of the image quality slices demonstrating the tools used to measure (a) contrast resolution, (b) spatial resolution and (c) subjective detail and noise. Figure 6 demonstrates the modulation transfer function (MTF) of the CT imaging system, generated by the Nichrome (80% nickel, 20% chromium) wire located in the lung region of slice 7.

### Phantom validation

Tables 2 and 3 contains both Table 2 CT number of tissue substitute materials measured inside the phantom and Table 3 results from the CSPT. Both bone and lung tissue used to construct the phantom were validated as equivalent to paediatric tissue across the indicated kV and filtration combinations. The soft tissue substitute material was validated for 70–80 kVp.

**Table 2:** Summary of results from phantom validation

Phantom material	Measured CT Number (HU) in phantom materials			
Rib substitute	700–970	610–860	485–700	350–525
Spine substitute	780–900	670–770	535–610	372–407
Soft tissue substitute	75–102	83–112	101–123	99–139
Lung substitute	− 760 to − 690	− 760 to − 690	− 760 to − 685	− 772 to − 690

**Table 3:** Summary of results from the clinical survey of paediatric tissues

	Group 1	Group 2	Group 3	Group 4
Tube voltage/filtration	70 kVp	80 kVp	100 kVp	Sn 100 kVp
No. of patients surveyed	14	16	14	17
Mean age of patients (years)	0.51	1.44	1.57	0.86

Tissue surveyed	Measured CT Number (HU) in paediatric tissues			
Rib	300–1600	300–1300	300–1000	200–900
Spine	200–1500	250–1300	200–1000	200–800
Soft tissue	20–100	10–130	30–90	20–80
Fat	− 150 to − 50	− 160 to − 60	− 140 to − 60	− 140 to − 50
Lung	− 900 to − 200	− 900 to − 400	900 to − 500	− 900 to − 400

### Clinical survey of paediatric tissues

**Fig. 7:**
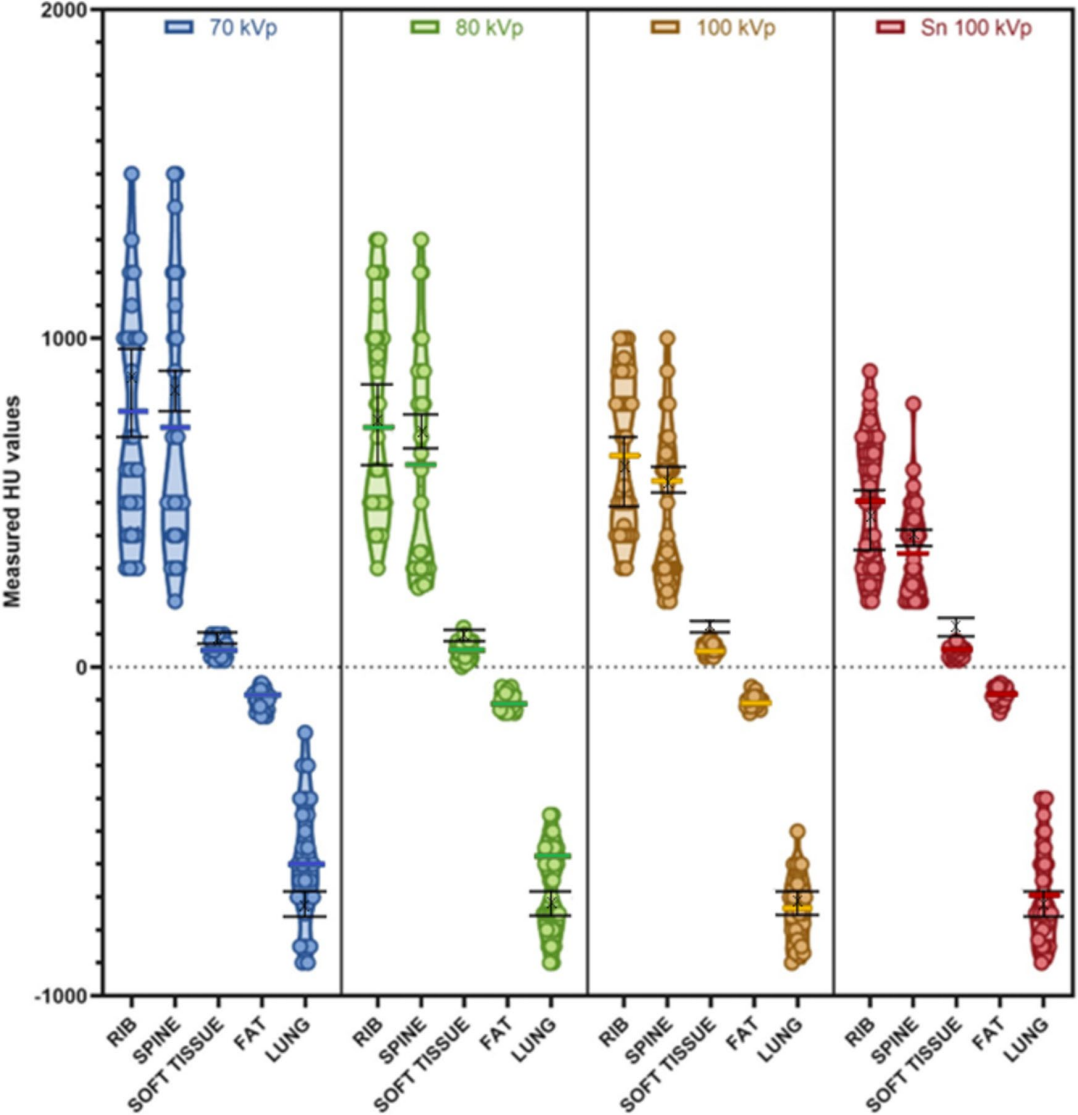
The coloured data illustrates results from the clinical survey of paediatric tissues (CSPT). Data is grouped by scanning tube voltage and filtration. Maximum and minimum CT numbers are plotted for paediatric tissues: rib, spine, soft tissue, fat and lung. Results from the validation of tissue substitute materials at corresponding beam qualities is overlayed in black

The clinically observed ranges of CT numbers measured in the CSPT are provided in Table 3 and visually depicted in Fig. 7. Each datapoint is either the maximum or minimum CT number recorded within a given structure for a given patient. Results are shown for five tissues [rib, spine, soft tissue, fat and lung], and four beam energies [70 kVp, 80 kVp, 100 kVp and Sn100kVp].

## Discussion

### Tissue substitute materials

PMMA is well-documented as a soft tissue equivalent at low diagnostic beam energies [17], but this agreement drifts at higher diagnostic beam energies, and also with added beam filtration (as seen in Tables 2, 3 and Fig. 7). Alternative materials have been used to model newborn thoracic tissues, for instance, Jamal et al. produced a low-cost newborn chest phantom for radiography, using beeswax [10]. Other studies have reported various plastics and wood that can be 3D printed for soft tissue substitutes in phantom construction for radiology [19] and nuclear medicine [20].

Eurosil-10 with added gypsum has demonstrated versatility in the field of phantom construction as it has successfully been used to model the radiological properties of adult bone in a radiotherapy phantom [16]. The resulting CT number is dependent on the amount of added gypsum and the CT beam quality. The data in Fig. 3 could be used to interpolate the concentration of gypsum required to model bony structures of adolescents or adults.

Figure 3 clearly demonstrates the increased CT numbers associated with bony structures and lower beam energies compared to higher beam energies and heavily filtered beams. This finding was further supported by the results from the CSPT (Fig. 7) where the maximum CT numbers observed in bony structures decreased as the beam energy and filtration increased.

Eurosil-4, the alternative soft tissue substitute material required for constructing the contrast resolution tool (slice 7) displayed significantly higher HU values compared to soft tissue across all beam energies and was not considered equivalent to soft tissue, as was initially desired. The contrast levels were made using added softener to decrease the density, and therefore the CT number but this was unsuccessful at producing distinctive contrast levels under clinical conditions. Muir and Laban produced a resolution tool, with contrast levels constructed by adding silicone blasting sand to a Pinkysil product, in a phantom designed for dental CBCT [21].

### Phantom design and construction

The phantom dimensions were consistent with the effective diameter of a 5-month-old child according to the AAPM lookup chart [18]. The phantom cost approximately $300 in materials.

The CNC milling machine is commonly used for fabrication of plastics. It was chosen due to it’s widespread availability, programmability and reproducibility. However, we encountered obstacles including limitations on geometric pathways associated with specific tool-bits and compatibility issues between Mastercam design file types and the available CNC software. This resulted in some deviation from the original design.

The addition of patient arms is of clinical relevance for paediatric CT, since infants are typically scanned in a cradle with their arms secured by their sides to reduce movement during imaging. The alternative central PMMA rods can be used to model paediatric arms, which are typically included in the scan range for patients under 6 months of age.

### Dosimetry and image quality slices

Figure 5 shows the reconstructed CT images of some of the phantom image quality slices. Slice 6 was used to measure contrast resolution in the lung equivalent tissue the iodinated foam produced contrast levels that could be visually distinguished from background in 4 progressive stages (Fig. 5a). With appropriate window and levelling, the contrast resolution tool can be used as a subjective measure of image contrast. Slice 5 was used for measuring contrast resolution in soft tissue equivalent material. Unfortunately, the Eurosil-4 Pink solution used to make this tool displayed a CT number between 320–370 HU at 70 kVp and was therefore not a desirable paediatric soft tissue substitute because it fell outside of the clinical range 20–100 HU at 70 kVp found in the CSPT. The increased amounts of softener were successful in decreasing the CT number but not significantly enough to be distinguished by human eye. For Slice 8, Nichrome wire inserted in the EVA foam background (Fig. 5b) a short script was written and executed via MATLAB Version 2011a (Natick, Massachusetts: The MathWorks Inc.) to generate the MTF plots displayed in Fig. 6. In slice 9, five progressively smaller air gaps can be viewed in the lung equivalent material of the contrast detail phantom slice (Fig. 5c). The resolution of these air gaps progressively worsens in the presence of noise and with decreasing slice thickness. The reverse pattern was designed to reduce any effects due to anatomical differences.

### Phantom validation

The soft tissue substitute material was considered equivalent to paediatric tissue when scanning at 70–80 kVp but not at 100 kVp, either with or without added tin filtration. In this case, the CT numbers from PMMA (101–123 and 99–139 HU) exceeded the range of CT numbers clinically observed (30–90 and 20–80 HU) in cardiac muscle of paediatric patients scanned at 100 kVp and Sn100 kVp.

The lung tissue substitute material, cork, was validated to be equivalent to paediatric lung tissue as it fell well within the clinically observed range of HU values across all beam qualities tested.

The bone tissue substitute materials were also validated as equivalent to paediatric bone for both ribs and spine according to the wide range of HU values observed in paediatric bone tissue in the CSPT.

### Clinical survey of paediatric tissues

Measuring and analysing average CT numbers within paediatric tissues was complex and not pertinent to this study. Instead maximum and minimum values were collected to classify a range of clinically observed CT numbers within paediatric tissues. It should be considered that all the patients included in this dataset had clinical pathologies or queries and some patient’s bone development could be inaccurate due to premature birth.

As demonstrated in Tables 2, 3 and illustrated in Fig. 7, the CSPT identified a range of clinically observed CT numbers in the thoracic structures of children across the four groups. The range of CT numbers differed the most in bony structures, while the minimum CT number remained relatively consistent, the maximum HU values observed in bone decreased significantly with increasing kVp and filtration. Very little difference in range was observed in the measure CT numbers of lung, fat and soft tissue when comparing different beam qualities. The overall range of CT numbers taken as the maximum of bone tissue compared to the minimum of lung tissue was the largest in the 70 kVp group and smallest in the Sn100 kVp group.

## Conclusion

A low-cost paediatric chest phantom was designed to approximate the geometrical and radiological properties of an infant’s thoracic structure. The tissue substitute materials were validated over a clinical range of energies appropriate for paediatric diagnostic CT. The phantom is equipped with utilities for dosimetry using either a farmer ionisation chamber or TLDs. The inclusive image quality tools allow assessment of spatial resolution, noise, contrast-to-noise ratio and subjective image quality measurements to be made. The phantom can be a valuable tool in the optimisation of paediatric chest CT.

## Data Availability

Additional data is available upon request via the corresponding authors.
